# Population-Genetic Inference from Pooled-Sequencing Data

**DOI:** 10.1093/gbe/evu085

**Published:** 2014-04-30

**Authors:** Michael Lynch, Darius Bost, Sade Wilson, Takahiro Maruki, Scott Harrison

**Affiliations:** ^1^Department of Biology, Indiana University, Bloomington; ^2^Department of Biology, North Carolina A&T State University

**Keywords:** allele-frequency estimation, population genomics, population subdivision

## Abstract

Although pooled-population sequencing has become a widely used approach for estimating allele frequencies, most work has proceeded in the absence of a proper statistical framework. We introduce a self-sufficient, closed-form, maximum-likelihood estimator for allele frequencies that accounts for errors associated with sequencing, and a likelihood-ratio test statistic that provides a simple means for evaluating the null hypothesis of monomorphism. Unbiased estimates of allele frequencies 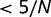 (where *N* is the number of individuals sampled) appear to be unachievable, and near-certain identification of a polymorphism requires a minor-allele frequency 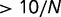. A framework is provided for testing for significant differences in allele frequencies between populations, taking into account sampling at the levels of individuals within populations and sequences within pooled samples. Analyses that fail to account for the two tiers of sampling suffer from very large false-positive rates and can become increasingly misleading with increasing depths of sequence coverage. The power to detect significant allele-frequency differences between two populations is very limited unless both the number of sampled individuals and depth of sequencing coverage exceed 100.

## Introduction

An increasingly popular approach to characterizing the genetic variation in a population involves pooling DNA from a large number of individuals into one sample from which a single DNA library is extracted. The sample is then sequenced to a high depth of coverage, with a goal of identifying the distribution of allele frequencies across the genome. Without individual tags, such a procedure eliminates the possibility of diploid-genotype identification, and except for sites close enough to be contained in the same sequence reads, there is also no possibility of linkage-disequilibrium estimation. Nonetheless, within certain constraints, pooled sampling has a number of potentially useful applications, for example, discovering single-nucleotide polymorphisms (SNPs), ascertaining the site-frequency spectrum within a population (i.e., the fraction of sites with different allele-frequencies), determining patterns of variation at various classes of sites (e.g., silent- vs. replacement-sites in protein-coding genes), and evaluating the amount of genetic differentiation among populations (including the identification of candidate markers associated with adaptive divergence) (Van Tassell et al. 2008; Futschik and Schlötterer 2010; Kofler et al. 2011; Boitard et al. 2012, 2013; Chubiz et al. 2012; Lamichhaney et al. 2012; Zhu et al. 2012; Gautier et al. 2013; Navon et al. 2013; Konczal et al. 2014; Lieberman et al. 2014).

However, the method of pooled-population sequencing introduces a number of statistical problems (Cutler and Jensen 2010), and an understanding of the limits of the approach is desirable. Some attempts have been made to derive estimators of summary statistics such as heterozygosity and population subdivision (e.g., Ferretti et al. 2013), but at least three issues remain unresolved. First, a statistically defensible allele-frequency estimator remains to be developed. The typical approach is to rely on arbitrary coverage cutoffs in inferring the validity of an SNP at a particular site, with the contributions from sequencing errors being dealt with in arbitrary or undisclosed ways. However, as will be demonstrated below, the observed frequency of raw reads at a site will generally yield a biased estimate of the true allele frequency. This can be especially problematical for rare alleles, which typically dominate polymorphic sites. Second, assuming that an appropriate allele-frequency estimator can be developed, it is unclear how the accuracy of estimation relates to the numbers of pooled individuals and the overall depth of sequence coverage for the sample. Although it is unlikely that a confident inference on the presence of an allele can be made if its frequency is less than the error rate, the actual cutoff for feasible SNP detection may be substantially greater than the error rate if the sample size is small. Finally, there is need for a formal basis for allele-frequency comparison across populations that accounts for the dual level of sampling that is unique to pooled sequencing (i.e., individuals within populations and sequences within pooled samples).

Here we present a maximum-likelihood (ML) estimator for the frequency of an allele in a pooled sample, taking into account the sampling strategy and factoring out the contribution from sequencing errors in a way that yields unbiased estimates with minimum sampling variance. After outlining the method, we use simulated data to evaluate false-positive rates associated with monomorphic sites (i.e., the false inference of a polymorphism encouraged by the presence of sequencing errors) and false-negative rates associated with polymorphic sites (i.e., the failure to detect a true polymorphism). Some “rules-of-thumb” will also be presented for identifying minimal detectable allele frequencies as a function of the error rate, sample size, and coverage. Finally, we will present a simple likelihood-based approach for detecting allele-frequency differences between populations, again evaluating its power as a function of the experimental setting.

## Allele-Frequency Estimation

We start with the assumption of a nucleotide site containing no more than two alleles with major-allele frequency *p* in the sample and an error rate ϵ per read. A biallelic model is justified by the extreme rarity triallelic variation at nucleotide sites, and in the unusual situation in which such a situation did exist, the frequencies of the two most common alleles and of the error rate would be slightly overestimated. Assuming each sampled nucleotide has a probability 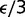 of being misread as any one of the alternative nucleotides, the probability that a random read is recorded as a major allele is
(1a)
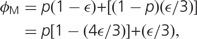

whereas the probability that the read is recorded as a minor allele is
(1b)
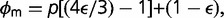

with the expected total fraction of reads corresponding to the two alternative (error) states being 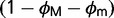.

Given a total coverage of 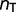 sequence reads at the site, which partitions to 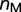 putative major, 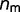 putative minor, and 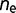 putative error reads (of the two alternative nucleotides), the likelihood of the observed data conditional on major-allele frequency *p* and error rate *ϵ* is then
(2)
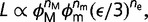

ignoring the trinomial coefficient, which is a constant independent of *p* and *ϵ* with no influence on the form of the likelihood function. This expression arises under the assumption that errors are random and equal in all directions, so that one-third of errors are to each of the alternative nucleotides, one of which may be a legitimate allelic state, that is, the major or minor allele. Taking the partial derivatives of equation (2) with respect to *p* and *ϵ* and setting them equal to zero yields the ML estimators of the error rate and major-allele frequency,
(3a)
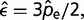

(3b)
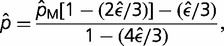

where 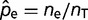 is the fraction of observed reads that are putative errors, and 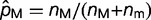 is the fraction of putatively nonerroneous reads that are of the candidate major type.

To evaluate whether an allele-frequency estimate is significantly greater than zero, we require the log likelihood of the data given the fitted model, which from equation (2) is
(4a)
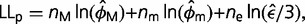

where 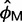 and 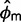 are defined as earlier with the ML estimates 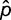 and 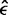 substituted for the parametric values. The log likelihood of the data under the assumption of monomorphism for the major allele is given by
(4b)
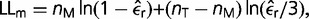

where 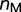 is the most abundant nucleotide read, and 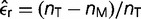. The likelihood-ratio test statistic,
(5)
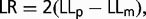

is then expected to be asymptotically 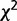-distributed with 1 degree of freedom (with cutoff values of 3.841, 6.635, and 10.827 for significance at the 0.05, 0.01, and 0.001 levels, respectively).

Two key issues are whether equation (3b) yields unbiased estimates of the allele frequency, that is, whether on average 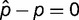, and whether the approach yields estimates with minimum sampling variance. A simple benchmark for the latter is derived by noting that pooled sequencing involves two levels of sampling: *N* individuals sampled from the population, and 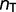 sequences subsequently extracted from the pooled DNA. The minimum achievable sampling variance of the allele frequency is then
(6)
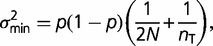

assuming diploidy (with *N* being substituted for 2*N* with haploidy or completely inbred lines). Note that even with infinite coverage, the expected sampling variance is no less than 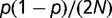, and little is gained in terms of precision by pushing the coverage per site much beyond 2*N*. Similarly, if the sample size substantially exceeds the coverage per site, the sampling variance is expected to asymptotically approach 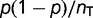, as nearly every read will be from a different chromosome.

**Fig. 1.—:**
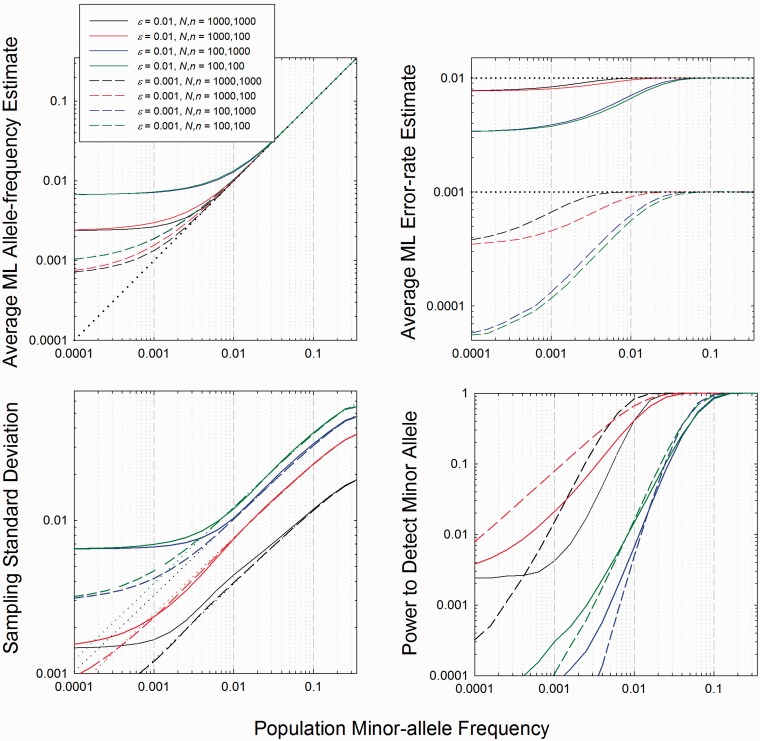
Performance of the ML estimator evaluated with simulation data. Upper left and right: Average estimates of the minor-allele frequency and the error rate using equations (3a) and (3b) for various numbers of individuals sampled (*N*), coverage per sequenced site (*n*), and error rate (ϵ); the diagonal line on the left and the horizontal lines on the right give the expected pattern in the absence of estimation bias. Lower left: Sampling standard deviation of the ML allele-frequency estimates; dotted lines are the theoretically minimum possible values, defined by equation (6). Lower right: The power to detect a minor allele at the *P* < 0.001 level with the likelihood-ratio test statistic.

To evaluate the performance of the estimator, we created simulated data sets, sampling *N* diploid individuals and then resampling the random pool for sequencing at depth-of-coverage *n*, assigning errors to each alternative nucleotide for each read with probability 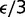. For each set of conditions, 500,000 simulations were done to obtain the mean and sampling variance of the ML estimates. For the range of sample sizes and error rates likely to be encountered in this sort of work, the ML estimator yields unbiased estimates of allele frequencies greater than roughly 5/*N* (fig. 1). At lower frequencies, the true allele frequency is overestimated, and the error rate is underestimated. This behavior occurs because when only two nucleotides are observed at a site, the ML estimator always interprets the rarer read as the minor allele, returning a zero error rate. When the true minor-allele frequency is on the order of *ϵ* or smaller, and the sample size is small, a large fraction of cases in which only two nucleotides are observed are ones in which the second most abundant nucleotide is simply an error (not the minor allele).

The results in figure 1 suggest a simple way to correct for the bias in allele-frequency estimates. After a first pass through a data set, one will have estimates of *p* and *ϵ* for the full set of sites. For the subset with significant major-allele frequency estimates 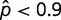, one can generally safely assume that the estimate 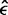 is unbiased, and an average value of 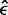 over all such sites will provide an estimate 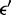 that can be back-applied to all sites for which 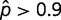 in the first round of estimation. That is, after substituting 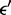, equation (3b) can be used to obtain essentially unbiased estimates of *p* for high-frequency alleles. Some of these estimates might slightly exceed 1.0, but that is an essential feature of an unbiased estimator.

The simulation results also show that the sampling standard deviation of the ML allele-frequency estimates is extremely close to the theoretical minimum defined by equation (6), provided the minor-allele frequency exceeds 0.01 for the conditions shown (fig. 1). Thus, as desirable for a sample statistic, the ML estimator yields asymptotically unbiased and minimum sampling variance estimates with increasing sample sizes and allele frequencies, and the deviations of theory from expectations will decline when the secondary modifications noted in the previous paragraph are implemented.

**Fig. 2.—:**
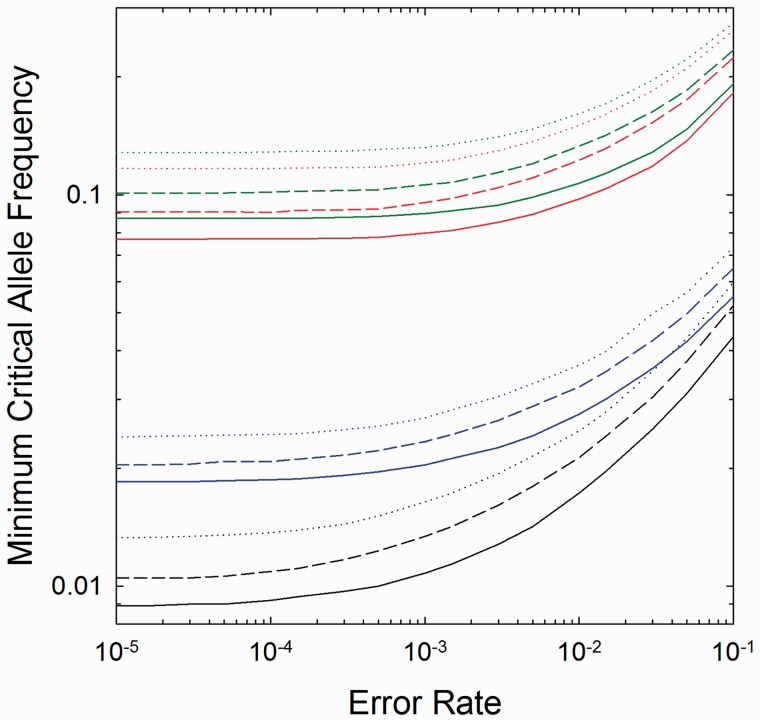
The critical minor-allele frequency within a population above which there is a 95% probability of detection with the likelihood-ratio test with significance levels set at 0.01 (solid lines), 0.001 (dashed lines), and 0.0001 (dotted lines). Color coding for sample sizes and error rates is the same as that given in figure 1.

Finally, we note that the power to detect a minor allele (1.0 minus the false-negative rate) increases with both the sample size and depth of coverage, and decreases with increasing error rate, as expected (fig. 1). If the significance cutoff level for detection by the likelihood-ratio test statistic is set at the *P* = 0.001 level, for the conditions shown, a minor allele must have a frequency in excess of 10/*N* to be detectable with near certainty, and even for a power to detect 10% of the time, the minor-allele frequency must exceed 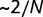. The false-positive rate, that is, the frequency at which the test is viewed as significant when the true value of *p* is 1.0 (minor-allele 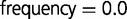), is generally well behaved, but can sometimes exceed the probability level of the statistical test. A somewhat different view is given in figure 2, which illustrates the minimum minor-allele frequency beyond which there is a high (95%) probability of detection with the likelihood-ratio test statistic, as a function of the error rate. Even with a negligible error rate, these critical values are on the order of 10/*N*, unless *n* < *N*, in which case they can be higher by 50% or so. Error rates on the order of 0.01 elevate the critical values by a factor up to 2-fold.

### Example

To evaluate the performance of the proposed allele-frequency estimator when applied to real data, we examined pooled-sequencing data for sites on chromosome 2L in library B6 produced by Zhu et al. (2012), using sequence files kindly provided by the first author. This library contained even portions of DNA from 92 *Drosophila* Genetic Reference Panel (DGRP) (Mackay et al. 2012) strains, distributed over two subsidiary libraries (B2 and B4), with the total pooled sequence of B6 yielding a 40× average depth of coverage per site. To obtain the site-specific quartets of nucleotide read counts in the B6 sample, we first made mpileup files of libraries B2 and B4 using SAMtools (Li et al. 2009), then extracted the read quartets with sam2pro (http://guanine.evolbio.mpg.de/mlRho/sam2pro_0.3.tgz, last accessed May 15, 2014), and finally combined the quartets for libraries B2 and B4. To avoid the use of potentially mismapped reads, we removed sites predicted to be in repetitive sequences (downloaded from ftp://ftp.ensembl.org/pub/release-65/fasta/drosophila_melanogaster/dna/, last accessed May 15, 2014) as well as those with coverage greater than twice the mean, leaving a total of 21,357,137 sites.

As benchmarks, the estimated allele frequencies at each site in the original DGRP data were calculated by extracting the nucleotides recorded for the genome sequences corresponding to the strains in library B6 (downloaded from http://www.hgsc.bcm.tmc.edu/projects/dgrp/freeze1, last accessed May 15, 2014). Unfortunately, genome sequences of only 85 out of the 92 strains were found in the DGRP database, and the number of sites with genotype data varied among strains due to variation in coverage. The final yield was 16,692,769 sites with allele-frequency estimates obtained both directly from the DGRP data and estimated from pooled-sequence data with our ML method. Of these sites, 15,948,891 were deemed monomorphic from the DGRP data.

**Fig. 3.—:**
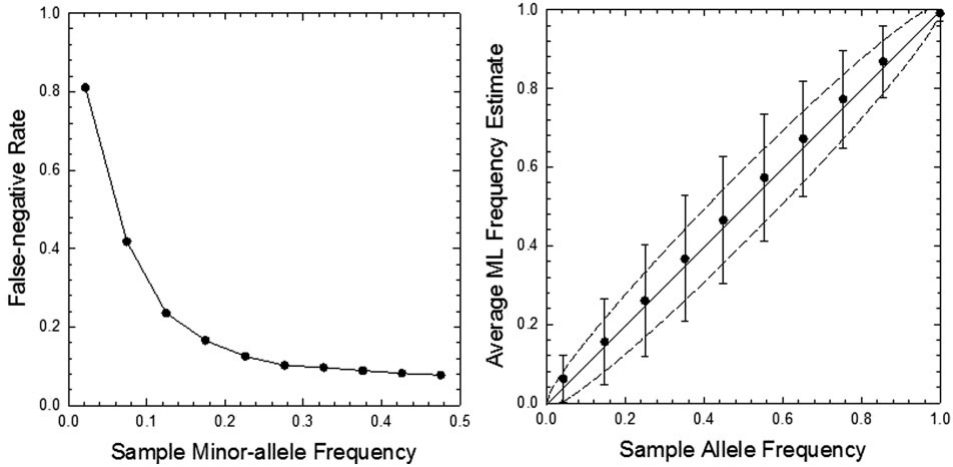
Left: False-negative rates (failure to detect at the 0.05 probability level) for bins of DGRP allele frequencies (obtained as described in the text). Right: Mean and standard deviations of ML estimates of binned DGRP allele frequencies. The diagonal line denotes positions of perfect correspondence, and the dashed lines denote single standard deviations above and below the expectation derived from equation (6) with 2*N* being set equal to the number of fly strains (ignoring diploidy because the lines were inbred) and 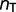 being set equal to the average depth of coverage (40).

Among the 15,948,891 monomorphic sites, the null hypothesis of monomorphism was rejected by the ML estimator at the 5% significance level at 641,457 sites. This suggests an overall false-positive rate of the ML estimator of 0.04, close to the expectation of 0.05, although the assumption here is that the DGRP data reflect the true situation. For the 743,878 sites deemed polymorphic from the DGRP data, the null hypothesis of monomorphism was accepted by the ML estimator at the 5% significance level at 373,673 sites, suggesting an overall false-negative rate of the ML estimator of 0.50, again assuming that the DGRP data themselves are correct. Not surprisingly, the false-negative rate is strongly influenced by the minor-allele frequency at a site, rapidly decreasing as the DGRP frequency increases, although still >10% even as the allele frequency approached 0.5 (fig. 3, left). On average, the ML estimates are very close to those derived directly from the DGRP data, consistent with the estimator being unbiased (fig. 3, right). The sampling standard deviations of the ML estimates somewhat exceed those predicted by equation (6). This may be a consequence of excess variation in sample size (*N*) and depth of coverage (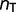) per site, which can result from variation in the amount of DNA associated with each genotype loaded into a pooled sample. However, some additional error is also expected to result from inaccuracies in the baseline DGRP allele-frequency estimates.

## Population Comparison

The preceding likelihood estimator (eqs. 3a and b) provides a convenient means of rapidly obtaining estimates of allele frequencies from pooled samples. However, although the likelihood given by equation (4a) continues to increase with increasing depth of coverage, this only provides increasing confidence in the sample estimate, not in the parametric value of allele frequency in the population itself (even though the sample estimate is an unbiased estimator of the latter). This issue becomes important when the goal is to compare allele frequencies in two different samples.

For purposes of statistical testing, we require a method that accounts for sampling of both individuals within populations and sequences within each pooled-population sample. This is accomplished by use of the following likelihood function:
(7)
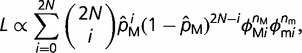

where 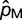 is the ML estimate of the major-allele frequency in the sample; 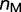 and 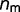 are the numbers of counts for major and minor alleles in the sample, respectively; and 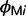 and 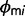 are defined as in equations (1a) and (1b) with 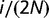 substituted for *p*. This expression approximates the total likelihood for a set of reads by summing over the probabilities of all possible samplings of the alleles from the population and accounting for the probability of the observed quartet given the sample. The trinomial coefficient defining the multiplicity of read counts and a term involving errors are ignored, as both remain constant in interpopulation comparisons and hence have no influence on the following statistical test.

To test for the significance of an allele-frequency difference between two samples, we first require the joint likelihoods of the observed reads in both samples starting with the assumption of population homogeneity. For such purposes, we start with summed quartets over both populations to obtain an estimate of the total major-allele frequency 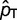 using equation (3b). Substituting this estimate for 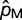 in equation (7), and using the major- and minor-allele counts in the first population (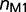 and 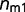), we then have an estimate of the likelihood of the observed quartet in population 1 under the assumption of population frequency 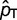, which we refer to as 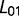. Likewise, using 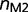 and 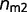 as the counts for the second population, the likelihood for the reads observed in population 2 under the null model is 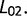 The likelihood of the quartet in population 1 under the full model (assuming population frequency heterogeneity) is obtained in the same manner, but by using the estimated allele frequency 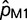 specific to this population (as well as 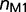 and 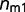), yielding 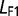, with similar treatment for population 2 yielding 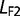. The likelihood-ratio test statistic for allele-frequency heterogeneity is then given by
(8)
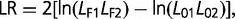

which is expected to be approximately 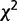-distributed with 1 degree of freedom.

**Fig. 4.—:**
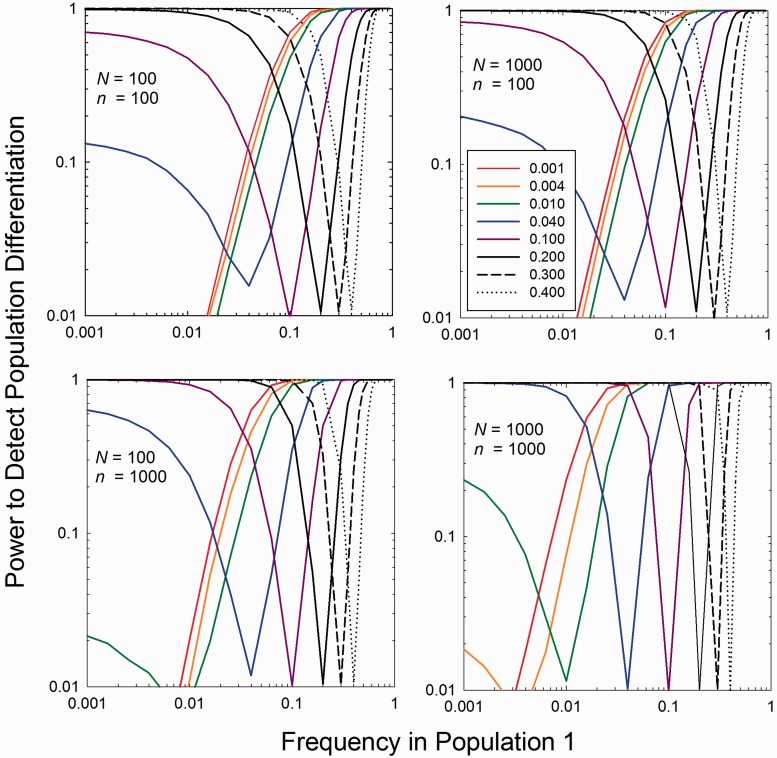
The power to detect a difference in allele frequencies between two populations at the *P* = 0.01 level. In each of the four panels, the number of diploid individuals sampled (*N*) and the sequencing coverage per sample (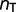) are assumed to be the same for both populations. For simulated data (50,000 data sets at each pair of frequencies), each line gives the fraction of times a difference was detected for a full range of allele frequencies in one population relative to a reference population with fixed frequency (given in the inset), using the 0.01 level of significance as a benchmark (one minus this probability is the false-negative rate, i.e., the probability of not detecting a difference when one exists). The false-positive rates (i.e., the probabilities of concluding that a difference exists when the two samples are from populations with identical frequencies) are equivalent to minima in the probability curves.

Application of this method to simulated data sheds light on the conditions under which allele-frequency differences can be detected (fig. 4). First, unless a rare allele in one population has a frequency exceeding several times 1/*N*, there is effectively no chance of detecting a difference between a population with a still lower frequency. Second, the power of detecting a difference in allele frequency is largely determined by the level of the survey with the smallest sample size. That is, the power for the situation in which a pool of *N* = 100 individuals is sequenced to 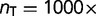 total coverage is not much different than that for a pool of 1,000 individuals sequenced to 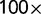 total coverage, nor even much different than the 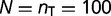 situation. Because sequencing is currently usually more expensive than sampling of individuals, this clearly implies that there is little advantage to sequencing at a depth of coverage much greater than the numbers of individuals in the pool—provided 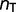 is on the order of *N* or smaller, essentially every sequence will be derived from a different chromosome. Third, even with very large sample sizes at both levels, there is effectively no power to detect a difference in which both populations have allele frequencies on the order of the error rate or smaller. Fourth, the test statistic behaves optimally in the sense that, for alleles with detectable frequencies, the false-positive rate is very close to the probability level of the corresponding evaluation level. This can be seen by referring to the positions in the figure in which the allele frequencies in both populations are identical. In all cases the false-positive rate is approximately 0.01, which is the significance level of the plotted power analyses.

### Example

As an example of the limited power of the experimental designs in recent comparative studies, consider the analysis of Burke et al. (2010), which compared a control with an experimental *Drosophila* population selected for rapid development. Pooling *N* = 125 individuals from each population, and then sequencing each of the two samples to 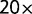 coverage, the authors detected 688,520 SNPs. They then focused only on the reduced set of 37,185 SNPs found at nonsynonymous sites in protein-coding genes, 662 of which were deemed to be significant at the 0.0001 level using a Fisher’s exact test for frequency differences. If the underlying assumptions of the statistical model were correct, this would lead to only 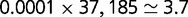 false positives in the final analysis, leading the authors to infer the presence of 658 candidate SNPs associated with the causative differences between the two populations.

**Fig. 5.—:**
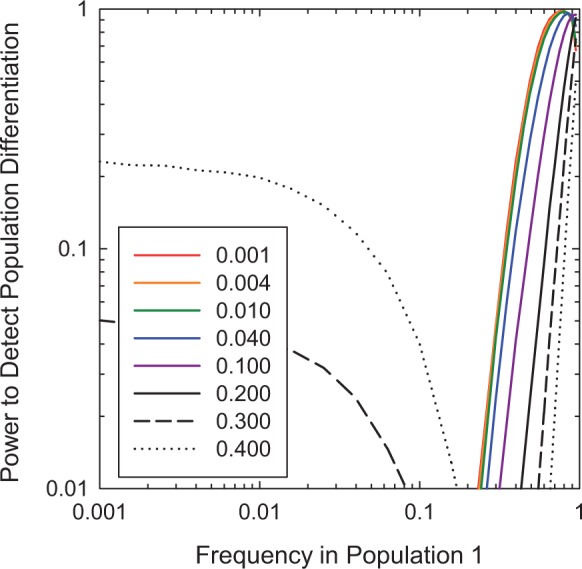
The power to detect differences in allele frequencies in the experiment of Burke et al. (2010), as in figure 4, but with *P* = 0.0001 as employed by the authors.

A central problem with this analysis is that the statistical test does not account for the two tiers of sampling noted earlier, and at increasingly higher levels of coverage, the authors would have concluded that more and more SNP frequencies differed significantly between the two samples even if they were invariant in the actual populations. Applying equations (7) and (8) with an assumed sequencing error rate of 0.01, the power of this experiment to detect significant differences at the 0.0001 level is illustrated in figure 5. To achieve even a relatively low power of detection of 50%, if an allele were completely absent from one population, the frequency in the other population would have to exceed 0.5. Similarly, if the actual frequency in one population were 0.1, that in the other would need to exceed 0.69 for a 50% power of detection; and if one frequency were 0.4, the other must exceed 0.95. In other words, over the entire frequency spectrum for this particular experiment, there is a <50% chance of detecting a frequency difference between populations smaller than approximately 0.5 using an appropriate statistical framework. Even for a 10% probability of detection, the critical difference in frequencies is approximately 0.4. This implies that, depending on the actual allele-frequency distribution, the number of candidate loci involved in differentiation of the two populations in this study must be substantially different than 658, most of the stated differences being a simple consequence of limited sampling (at most 20 alleles per sample).

It is also worth noting that prior to analysis the two sets of populations were maintained for 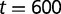 generations at total population sizes of approximately *M* = 1,000 individuals. Because effective population sizes are typically much smaller than actual population sizes, this means that the standard deviations of allele-frequency changes for purely neutral loci must substantially exceed 
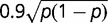
, where *p* is the initial allele frequency and the 0.9 is obtained from the expectation for the cumulative amount of drift, 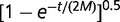, so substantial differences in these populations will have arisen by random genetic drift alone. Thus, given the design of the overall experiment, it is difficult to conclude anything about the causal source of divergence. Notably, in a full scan of the genome, the authors found no evidence of selective sweeps associated with the fixation of advantageous alleles although there were some regions with apparent heterozygosity reduction.

Numerous other studies involving pooled comparisons have utilized sampling strategies similar to that noted earlier. For example, in a study involving a north–south cline in *D. melanogaster*, Kolaczkowski et al. (2011) relied on 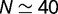 and 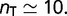 The authors qualitatively inferred numerous chromosomal regions of interest based on measures of population subdivision, although because of the lack of information on the amount of divergence expected via genetic drift alone, interpretation of the observed results is difficult. In another “select-and-sequence” study, Turner et al. (2011) studied body-size differentiation in *D. melanogaster* populations with *N* = 75 and 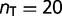, and after accounting for the contributions of random genetic drift, concluded that 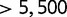 SNPs had diverged in frequency as a consequence of selection, while acknowledging that the study was unable to evaluate the behavior of low-frequency alleles. Similarly, in an experiment involving divergent selection for courtship-song structure in *D. melanogaster* with *N* = 120 and 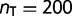, Turner and Miller (2012) concluded that thousands of SNPs changed in frequency by approximately 2.5%. Although the number of changes exceeded that expected after accounting for the expected contribution from genetic drift, no single variant exhibited a significant change.

## Discussion

The statistical procedures outlined above provide a logical framework for extracting population-genetic information from high-coverage genomic sequences derived from pooled-population samples. The method for allele-frequency estimation is efficient in terms of computational speed, allows for site-specific error rates, and yields estimates that are unbiased with minimal sampling variance (within the bounds dictated by the sampling scheme). With appropriate attention to error-rate estimation, as described earlier, it may be possible to obtain estimates of allele frequencies somewhat lower than the error rate, provided the population sample size and coverage are adequately large. The method for evaluating population differences is also statistically well behaved and accounts for sampling at both the population and sequencing levels.

Evaluation of the behavior of the likelihood statistics provides several insights into the limitations of pooled sequencing. First, to achieve a very high level of confidence in an allele-frequency estimate, the population-level frequency needs to exceed approximately 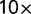 the reciprocal of the number of individuals sampled, for example, a minor-allele frequency of 0.1 for a sample size of 100. Second, unless the sample sizes at the population (*N*) and sequencing (*n*) levels are both substantially exceed 100, the power to detect differences in population frequencies is limited. Third, for fixed depth of sequence coverage (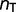), little is gained in terms of statistical power by pooling many more individuals than 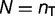.

Finally, we note that one practical issue that requires attention in any pooled-population analysis is the need to equilibrate the concentrations of DNA from each individual contributing to a pooled sample. The allele-frequency estimators that we provide are unlikely to biased in the face of unequal molar concentrations unless there is an association between particular nucleotide variants and the sizes of individuals. However, the sampling variance of the estimates will be inflated by unequal representation as the effective sample size would be smaller than the actual number of individuals in the pool.
